# The remarkable genetic relationship between *Staphylococcus aureus* isolates from hemodialysis patients and their household contacts: Homes as an important source of colonization and dissemination

**DOI:** 10.1371/journal.pone.0267276

**Published:** 2022-04-19

**Authors:** Daniela Montoya Urrego, Johanna M. Vanegas, J. Natalia Jiménez

**Affiliations:** 1 Grupo de investigación en Microbiología Básica y aplicada (MICROBA), Escuela de Microbiología, Universidad de Antioquia, Medellín, Colombia; 2 Grupo de investigación en Salud Pública, Escuela de Ciencias de la Salud, Universidad Pontificia Bolivariana, Medellín, Colombia; The Rockefeller University, UNITED STATES

## Abstract

**Introduction:**

*Staphylococcus aureus* is a successful pathogen in hospital and community. Hemodialysis patients have high colonization rates. Interactions between them and their household contacts, are an opportunity to understand the *S*. *aureus* colonization between hospitals and community. This study aims to determine the clinical and epidemiological characteristics of *S*. *aureus* colonization in hemodialysis patients and their household contacts, as well as the genetic relationship between their isolates.

**Methods:**

A cross-sectional study was conducted on hemodialysis patients from hospital-associated dialysis center in Medellín-Colombia, and their household contacts between 2019 and 2020. Colonization was assessed in the nostrils for household contacts and nostrils and skin around the catheter insertion for hemodialysis patients. Epidemiological information was obtained, and colonization was evaluated in their pets’ oral cavities. Bacterial identification and susceptibility were assessed using phenotypic and molecular methods. Molecular typing included SCC*mec* typing, pulsed-field gel electrophoresis (PFGE), *spa* typing, and virulence factor detection.

**Results:**

Colonization frequency was 35.6% (n = 16/45) in patients (87.5% MSSA– 12.5% MRSA) and 43.1% (n = 53/123) in household contacts (88.7% MSSA—11.3% MRSA). Of 45 homes, 77.8% presented colonized people. Colonization was detected in at least two household members in 46.7% of homes, of which 52.4% had a genetic relationship. Colonization was 16% (n = 4/25) in pets (75% MRSA—25% MSSA). The most frequent clonal complex was CC8 (15.6%), and the *spa* typing revealed high diversity.

**Conclusion:**

This study shows a high frequency of colonization by *S*. *aureus* in both hemodialysis patients and their household contacts and a significant genetic relationship between their isolates. This demonstrates an exchange of this bacterium and that homes are an important source of colonization to patients, highlighting the need for prevention strategies outside the hospital to avoid future infections, and the importance of the populations with permanent transit between the two environments.

## Introduction

Colonization by *Staphylococcus aureus* is considered an important risk factor for the development of skin and soft tissue infections and invasive infections in different groups of patients. Hemodialysis patients present comorbidities and specific clinical characteristics that favor high frequencies of colonization, even greater than those reported in hospitalized patients [1, 2]. Colonization by *S*. *aureus* in hemodialysis patients has serious implications due to its relationship with the development of endogenous infections, especially when colonization occurs persistently, as reported in a previous study that found that 77.8% of hemodialysis patients who developed *S*. *aureus* bacteremia were previously colonized by this microorganism [3]. Additionally, colonization in these patients increases the risk of the spread of the microorganisms both in health institutions and community because they can act as asymptomatic reservoirs and transporters of these bacteria for long periods, facilitating their transmission to people with whom they closely interact, such as their relatives and contacts within the community [4, 5].

In this way, hemodialysis patients and their household contacts are a model population to understand the dynamics of the dissemination of MSSA and MSRA between the hospital and the community because they constantly circulate between the two environments [4]. Also, the household contacts have a direct link of care and accompaniment with patients, which is why they share spaces, objects, and habits that can facilitate the transmission of bacteria among them [2]. At the same time, social, economic, epidemiological, cultural, and environmental factors converge in this population, which can favor the colonization by resistant bacteria from other environments than the hospital.

Taking into account that in Colombia, and particularly in Medellín, colonization by *S*. *aureus* in hemodialysis patients has been reported at 50.9% [6], in this study, it was proposed to determine the clinical and epidemiological characteristics of *S*. *aureus* colonization in hemodialysis patients and their household contacts, as well as the genetic relationship between their isolates, to establish and identify opportunities to prevent colonization and future infections in patients from non-hospital environments.

## Materials and methods

### Study population

A cross-sectional study was conducted between June 2019 and May 2020 with hemodialysis patients and their household contacts. The hemodialysis patients belong to a hospital-associated renal unit in Medellín, which treats approximately 350 patients. The study protocol was approved by the Bioethics Committee for Human Research of the University of Antioquia (CBEIH-SIU) on May 3, 2017 (approval no. 17-65-689), and written informed consent was obtained from each subject, or by their parents or legal guardian. As an inclusion criterion, patients had to share residence with at least one person; a household contact was defined as whom live in the same house as the patient for a period not less than six months. The exclusion criteria were hemodialysis patients living alone or refusing informed consent.

### Data collection

Hemodialysis patients were contacted from a cohort of a previous study in our research group, who presented previous colonization [3]. A visit to each home was agreed and, once informed consent had been accepted, the epidemiological and clinical information was obtained using a questionnaire designed for this purpose. The information included general and clinical information, hand washing-practice, and information about sharing some elements and spaces between household contacts and patients. Finally, any special care provided to the patients, such as help with personal hygiene, was taken into account. In addition, if there were pets in the home, information and clinical history were included.

### Colonization, identification, and susceptibility testing for *S*. *aureus*

For screening *S*. *aureus* colonization, samples were obtained with a sterile cotton swab with 0.9% sterile saline solution, from the nostrils of household contacts, and the nostrils and skin around the catheter insertion (if the central venous catheter was still used in treatment and had not switched to shunt) for patients. In the case of pets, samples were obtained from oral cavities in a subsequent visit (Approved by the ethics committee for experimentation with animals of the University of Antioquia (CEEA) with the minutes of session No. 136). Each participant’s sample was taken only once, however, all samples from the same home were taken simultaneously. Each swab was placed in AMIES (transport medium with activated carbon) and subsequently were enriched in trypticase soy broth (TSB) overnight at 37°C and then plated into mannitol salt agar, for the selection of indicative fermenting colonies of *S*. *aureus*. Preliminary identification was conducted by standard laboratory methods based on colony morphology in sheep blood agar and positive catalase and coagulase tests. The identification of isolates and antibiotic susceptibility was determined using the automated Vitek-2 system (bioMérieux) according to Clinical and Laboratory Standards Institute (CLSI) cutoff points [7].

### Molecular typing

DNA was extracted from the isolates using the Wizard Genomic DNA purification kit (Promega, Madison, USA) according to the manufacturer’s instructions. Molecular detection of the species-specific *nuc* gene and *mecA* gene (determinant of methicillin resistance) was verified by standardized polymerase chain reaction (PCR) [8, 9]. SCC*mec* types and subtypes were determined using multiplex PCR reactions [9, 10]. Similarly, the virulence genes that encode for Panton-Valentine Leucocidine (PVL), staphylococcal enterotoxins (*sea*, *seb*, *sec*, *sed*, *see*), exfoliative toxins A and B (*eta*, and *etb*), and toxic shock syndrome toxin 1 (*tsst*-1) were detected [11, 12].

In the event that at least two household members were colonized in the same home, the genetic relatedness of isolates from hemodialysis patients, household contacts, and pets was determined using pulsed-field gel electrophoresis (PFGE), which was performed using the *SmaI* restriction enzyme (Thermo Scientific, United States) [13]. The cluster analysis was performed using BioNumerics software version 6.0 (Applied Maths, SintMartens-Latem, Belgium), and dendrograms were generated by the unweighted pair group method using average linkages (UPGMA), with 1% tolerance and 0.5% optimization settings. A similarity cutoff of 80% was used to define genetically related strains.

Additionally, the amplification and sequencing of the polymorphic X region of protein A gene (*spa*) was conducted for all isolates, and corresponding *spa*-types were assigned using the *spa*-typing website www.spaserver.ridom.de/. Clonal complexes (CC) were inferred by *spa* repeat pattern analysis or by referring to the Ridom SpaServer website [14].

### Statistical analysis

Categorical variables were described as absolute and relative frequencies. Continuous variables were expressed as mean and standard deviation or median and interquartile range, according to the assumption or not of the normality. To determine factors potentially associated with colonization, a generalized linear model (GLM) was performed for a binomial distribution with a clog-log link function. Each exposure was analyzed in a different model and adjusted by age, sex, crowding index (number of people in home divided by number of rooms), and dwelling type, as previously suggested in literature [15]. Measures of association (prevalence ratios—PR) were expressed with their corresponding 95% confidence intervals (95% CI) and *p*-value. Statistical analyses were carried out using STATA software, version 14.0.

## Results

**Fig 1:**
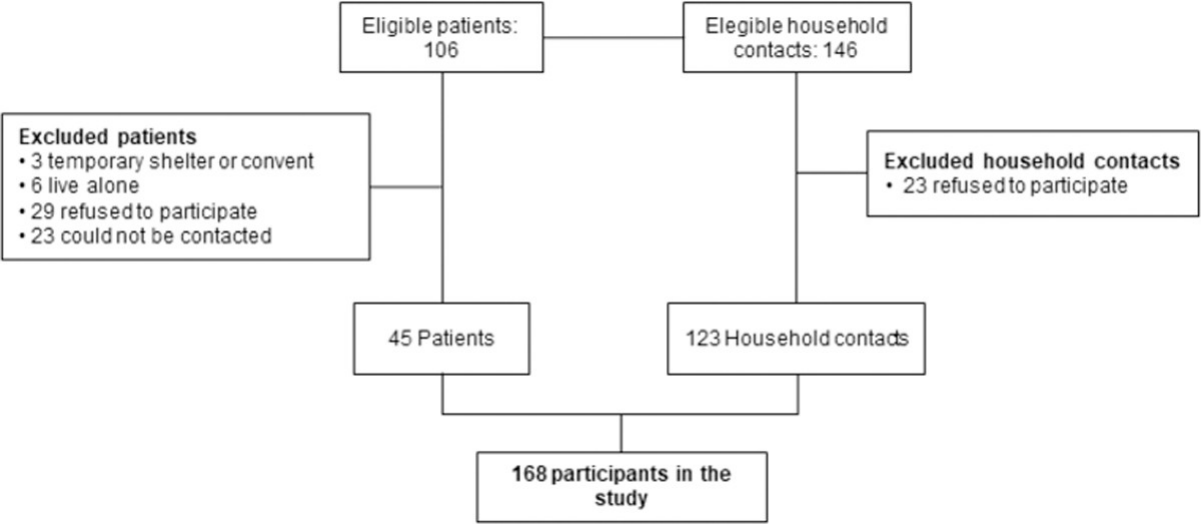
Inclusion of hemodialysis patients and their household contacts.

A total of 168 participants were enrolled, 45 patients and 123 household contacts. A flowchart illustrating the inclusion of participants is shown in Fig 1. All the participating families live in the urban area, the majority in Medellín (93.3%, n = 42), just 6.7% (n = 3) of the families live in other municipalities. The participants live in houses with an average of 3.5 rooms and 1.6 bathrooms. Furthermore, 40% (n = 18) of the patients had four or more households contacts.

Regarding to hemodialysis patients, in 30 (66.6%) swab samples were obtained from nostrils and skin around the catheter insertion, while in 15 patients (33.3%) only samples from nostrils were obtained, because they had changed the treatment to shunt. Most of the patients were men (51.2%, n = 23), and the median age was 62 years old (IQR 49–71.5). The clinical history revealed that 57.8% (n = 26) of the patients had been hospitalized in the last year, and the antibiotic consumption was 53.3% (n = 24). The most frequent comorbidities were arterial hypertension (84.4%, n = 38) and diabetes mellitus (40%, n = 18). Regarding their hand hygiene habits, most patients wash their hands before preparing food (60%, n = 27), after toilet (93.3%, n = 42) and before eating (77.8%, n = 35). However, 4.4% (n = 2) of patients claimed not to wash their hands at any time (Table 1).

**Table 1:** The clinical and epidemiological characteristics of hemodialysis patient and their household contacts according to colonization status by *S*. *aureus*.

Characteristic	Hemodialysis patients (n = 45)		Household contacts (n = 123)	
	Colonization n = 16 (35.6%) n (%)	Non-Colonization n = 29 (64.4%) n (%)	Colonization n = 53 (43.1%) n (%)	Non-colonization n = 70 (56.9%) n (%)
**Sex** (female)	8 (17.8)	14 (31.1)	34 (27.6)	47 (38.2)
**Age** Me (IQR)	56.5 (44.8–70)	63 (54.5–73)	39.5 (17–58.3)	35.5 (17.8–55.3)
**Smoking**				
Past smoking	4 (8.9)	9 (20)	11 (8.9)	12 (9.8)
Current smoking	0 (0)	2 (4.4)	2 (1.6)	5 (4.1)
**Comorbidities**				
Arterial hypertension	13 (28.9)	25 (55.6)	10 (8.1)	10 (8.1)
Diabetes mellitus	7 (15.6)	11 (24.4)	6 (4.9)	6 (4.9)
Heart disease	5 (11.1)	9 (20)	1 (0.8)	1 (0.8)
Dyslipidemia	4 (8.9)	9 (20)	2 (1.6)	4 (3.3)
**History in the last year**				
Home care services	5 (11.1)	11 (24.4)	1 (0.8)	11 (8.9)
Hospitalization	10 (22.2)	16 (35.6)	3 (2.4)	6 (4.9)
Surgeries	7 (15.6)	15 (33.3)	4 (3.3)	11 (8.9)
Antibiotics use	7 (15.6)	17 (37.8)	12 (9.8)	24 (19.5)
**Hand hygiene habits**				
Before cooking	9 (20)	18 (40)	39 (31.7)	59 (48)
Before eating	13 (28.9)	22 (48.9)	45 (36.6)	46 (37.4)
After eating	9 (20)	18 (40)	37 (30.1)	41 (33.3)
After toilet	15 (33.3)	27 (60)	51 (41.5)	64 (52)
When they get home	10 (22.2)	15 (33.3)	42 (34.1)	49 (39.8)
After changing a diaper	4 (8.9)	6 (13.3)	9 (7.3)	27 (22)
After playing with pet	4 (8.9)	9 (20)	17 (13.8)	33 (26.8)
Do not wash their hands	0 (0)	2 (4.4)	0 (0)	0 (0)

Most of the household contacts were women (65.8%, n = 81), and the median age was 37 years old (IQR 18–56). The relationships they had with the hemodialysis patient were of children (35%, n = 43), partners (17.9%, n = 22), and 47.2% (n = 58) parents or others. In the last year, 7.2% (n = 9) of them were hospitalized and 12.2% (n = 15) underwent some surgery. Most of them did not report any comorbidity (51.2%, n = 63); however, 16.3% (n = 20) and 9.8% (n = 12) reported arterial hypertension and diabetes mellitus, respectively. Regarding their hand hygiene habits, 93.5% (n = 115), 79.7% (n = 98) and 74% (n = 91) of them wash their hands after toilet, before preparing food and before eating, respectively (Table 1). About relationships and interaction between household contacts and patients, 75.6% (n = 93) shared the bathroom with the patient and 37.4% (n = 46) shared the bathroom soap, mainly bar soap (35%, n = 43). Other characteristics of the participants are described in Table 1.

### *Staphylococcus aureus* colonization

Of 45 homes, 77.8% (n = 35) presented colonized people, and in 46.7% (n = 21), colonization was detected in two or more household members. In 13 homes (28.8%), colonization by *S*. *aureus* was detected in hemodialysis patients and household contacts. In 19 homes (42.2%), only household contacts were colonized, and in 3 (6.7%) homes, only patients. Additionally, of 15 homes with pets, 3 (20%) had colonized animals. In all of them, there was at least one colonized person.

In general, colonization by *S*. *aureus* was 41.1% (n = 69), mainly by MSSA (88.4%, n = 61). In the hemodialysis patients, colonization was present in 35.6% (n = 16; 87.5% (n = 14) MSSA and 12.5% (n = 2) MRSA). On the other hand, in household contacts, colonization was present in 43.1% (n = 53; 88.7% (n = 47) MSSA and 11.3% (n = 6) MRSA). No distinction was observed between the colonization frequency presented in patients and their household contacts (p = 0.368). Additionally, a total of 25 pets were evaluated in the study (10 canines and 15 felines), colonization was found in 16% (n = 4, 75% (n = 3) MRSA and 25% (n = 1) MSSA), 2 cats and 2 dogs.

### Susceptibility, virulence factors, and molecular typing

**Fig 2:**
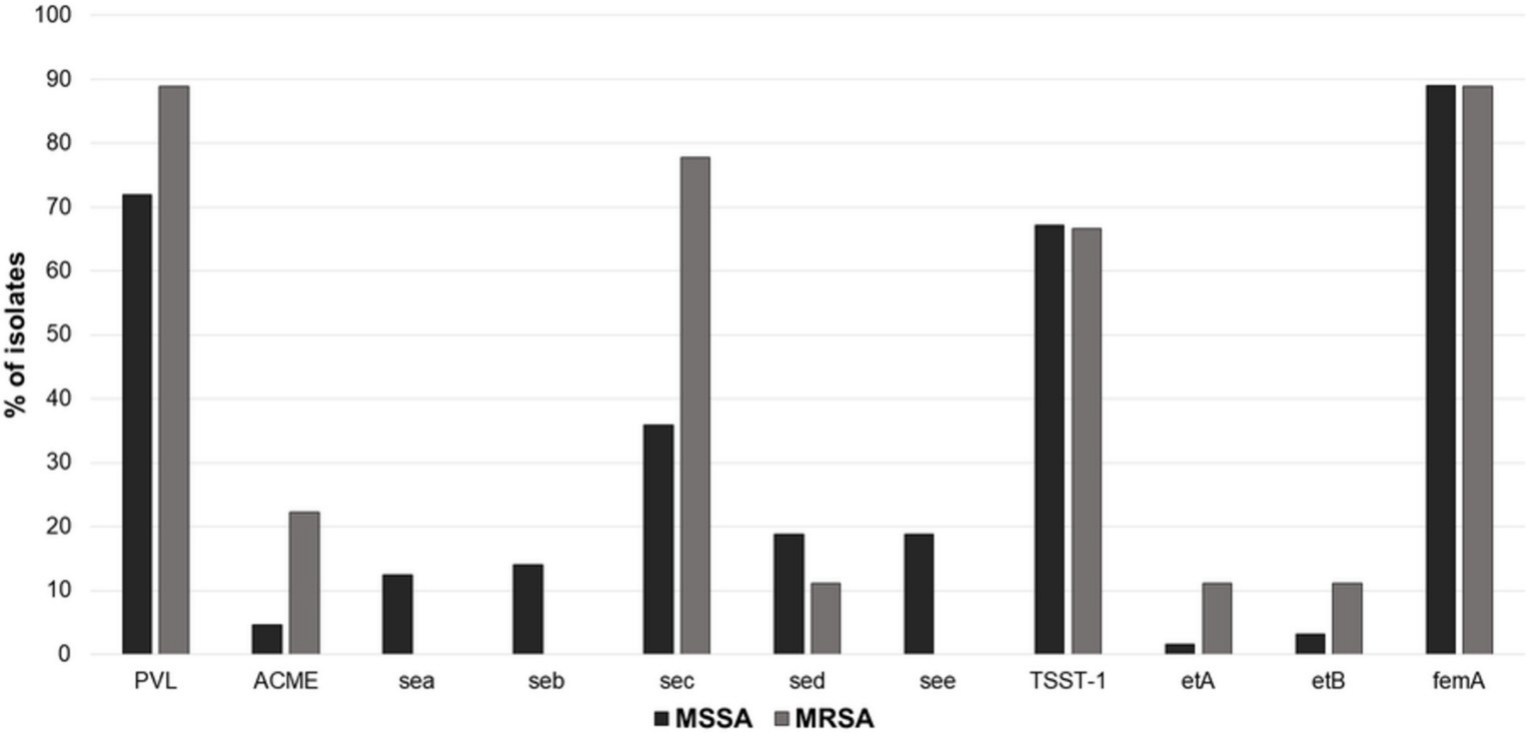
Frequency of virulence factors of *S*. *aureus* isolates. (**PVL:** Panton-Valentine Leucocidin; **ACME:** arginine catabolic mobile element; **SEA, SEB, SEC, SED, SEE:** staphylococcal enterotoxin genes A-E; **TSST-1:** toxic shock syndrome toxin 1; **ETA** and **ETB:** exfoliative toxin genes A and B; **FemA:** amino acyltransferase *femA* gen).

Of 12 MRSA isolates, 66.7% (n = 8) were resistant to tetracycline. Sixty-five MSSA isolates were evaluated, which showed resistance mainly to erythromycin (20%, n = 13), and clindamycin (15.4%, n = 10). Regarding the virulence factors, in both MSSA and MRSA isolates, the most frequently detected were PVL (71.9% and 88.9%, respectively), TSST-1 (67.2% and 66.7%, respectively), and enterotoxin C (35.9% and 77.7%, respectively). Among MSSA isolates, all the evaluated virulence factors were detected; however, enterotoxins A, B and E were not detected in the MRSA isolates (Fig 2).

In total, 39 *spa*-types were identified. The most predominant *spa*-types were t201 and t1236 (S1 Table). Eleven clonal complexes were inferred, and the most frequent were CC8 with 15.6% (n = 12), and CC30 with 14.3% (n = 11). All MRSA isolates (n = 12) were confirmed by the presence of *mecA* gene. The SCC*mec* type IVc was present in two isolates, and the SCC*mec* type IVb was present in one isolate.

**Fig 3:**
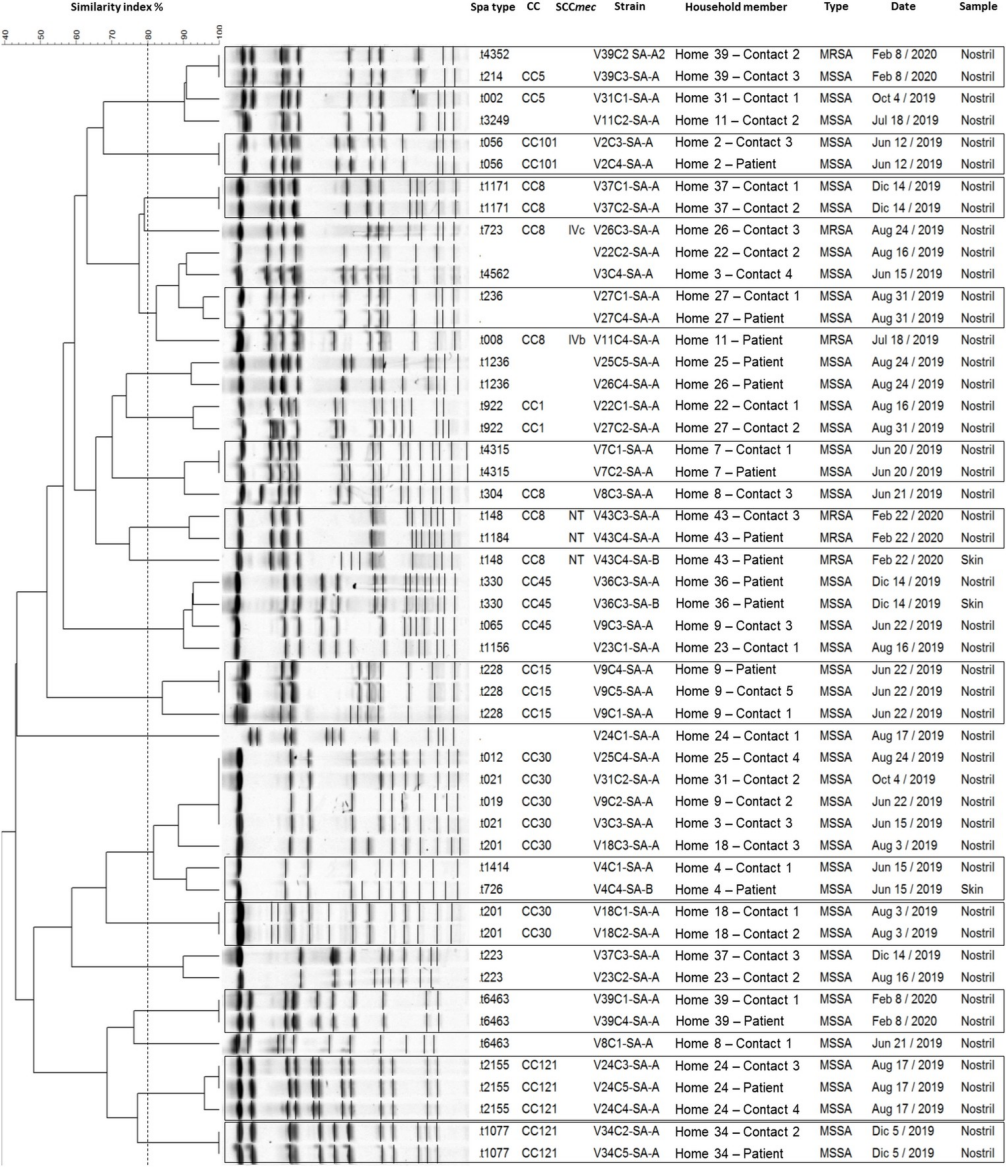
The genetic relatedness between *S*. *aureus* isolates colonizing hemodialysis patients and their household contacts. The isolates genetically related from the same home are boxed.

The analysis of pulsed-field electrophoresis was carried out in 21 homes, where at least two participants colonized by *S*. *aureus* were found. A high genetic relationship was found between isolates from different household members in 11 homes (52.4%); of them, in nine (81.8%), the genetic relationship occurred between the isolates of the patients and at least one of their household contacts (Shown in Fig 3). No genetic relationship was found between isolates from pets and someone in homes.

### Factors associated with colonization

The findings of the analysis of factors associated with colonization are presented in Table 2. The frequency of colonization was higher in household contacts that shared a towel with the hemodialysis patient (PR: 2.33; CI: 1.16–4.68; p = 0.017) and in homes where the crowding rate was positive (PR: 1.31; CI: 0.99–1.74; p = 0.059). On the other hand, home care service was shown as a protective factor for colonization by *S*. *aureus* (PR: 0.40; CI: 0.17–0.93; p = 0.034). In multivariate analysis, the only factor that maintained its association with colonization after adjustment by age, sex, crowding index, and dwelling type was home care service (PR: 0.41; CI: 0.18–0.97; p = 0.042). Other factors such as hospitalization and antibiotic use were not associated with colonization (Table 2).

**Table 2:** Bivariate and multivariate analysis for identification of factors associated with colonization by *S*. *aureus*.

Variable	Bivariate analysis				Multivariate analysis			
	PR	95% CI		p-Value	PR	95% CI		p-Value
Age	1.00	0.99	1.01	0.580	1.00^a^	0.99	1.01	0.985
Type of participant (Hemodialysis patient /Household contact)	0.78	0.44	1.37	0.387	0.74^b^	0.39	1.42	0.368
Crowding rate	1.31	0.99	1.74	0.059	1.31^c^	0.96	1.79	0.085
Current smoking	0.46	0.11	1.90	0.284	0.42^b^	0.10	1.77	0.237
Share towel with Hemodialysis patient	2.33	1.16	4.68	0.017	1.13^b^	0.65	1.96	0.662
Share bar soap with hemodialysis patient	1.26	0.72	2.20	0.409	1.20^b^	0.68	2.13	0.520
History in the last year								
Home care services	0.40	0.17	0.93	0.034	0.41^b^	0.18	0.97	0.042
Hospitalization	0.85	0.46	1.56	0.600	0.89^b^	0.47	1.66	0.709
Surgeries	0.69	0.37	1.28	0.240	0.72^b^	0.37	1.38	0.322
Antibiotics use	0.67	0.40	1.13	0.136	0.72^b^	0.42	1.23	0.227
Use of penicillin	0.89	0.42	1.88	0.769	0.98^b^	0.46	2.10	0.958
Use of beta-lactams	1.09	0.59	2.01	0.780	1.15^b^	0.62	2.13	0.663
Diabetes mellitus	1.09	0.59	2.01	0.780	1.20^b^	0.60	2.40	0.602

Abbreviation: CI, confidence interval; PR, Prevalence ratio

^a^ Adjusted by sex and crowding rate

^b^ Adjusted by age, sex, and crowding rate

^c^ Adjusted by age, sex, and dwelling type.

## Discussion

This study starts from colonization in a high-risk population such as hemodialysis patients, and tries to get closer to understanding the role that they and their household contacts have in the dynamics of *S*. *aureus* dissemination between the community and hospital, given that this population is constantly moving between the two environments.

The high genetic relationship found between isolates from different household members shows that homes are one of the main reservoirs of *S*. *aureus*, which is crucial to its success as community pathogen and is decisive in the colonization of patients. These results are similar to those described by Nouwen et al., where 65% of people colonized by *S*. *aureus* who lived in the same home shared genetically identical isolates [16]. By behaving as a source of colonization where household members share the bacteria, homes can increase the possibility of persistent colonization in this population [17]. This is of particular concern for hemodialysis patients, as being colonized increases their risk of developing endogenous bacteremia [3].

Likewise, the non-relationship observed in 47.6% of the isolates is similar to that reported in other studies, where the diversity of *S*. *aureus* strains circulating in homes ranged between 39% and 50% [18, 19]. This may correspond to acquisition of different strains from other sources in the community, which are subsequently taken into the homes [20]. Due to the ability of *S*. *aureus* to survive on surfaces and its capacity for person-to-person dissemination, non-hospital environments are potential reservoirs, such as schools, workplaces, supermarkets or public transport [21, 22]. Also, particular populations such as outpatients, health workers, and medical students play a fundamental role in the insertion and transport of bacteria from hospital environments to the community and vice versa [23, 24]. These different sources of colonization may explain the great diversity of isolates found in household contacts compared with hemodialysis patients, due to the possibility that their colonization occurs mainly from community spaces instead.

The frequency of colonization by *S*. *aureus* observed in the household contacts was higher compared to that observed in hemodialysis patients. However, this could be affected by the high consumption of antibiotics in patients. But it should be noted that in most homes, colonization was only found in the household contacts, and the frequency observed was higher compared to that observed in general population (17.6%– 32.4%) [25, 26]. Thereby, Zafar et al. reported that contacts of patients with previous *S*. *aureus* infection had a higher frequency of colonization than that reported in the general population, reinforcing the idea of the existence of a common source of exposure among them [18]. The frequency of MRSA colonization in our study was also higher compared to that described in general population without associated risk factors (1.3%) [27]. However, in a study carried out in dialysis patients, renal unit workers, and their relatives, it was observed that MRSA colonization does not exceed 4.0% [4]. This suggests that MRSA transmission may occur outside the hospital. This is supported by other factors such as easy access to antibiotics, self-medication, and lack of adherence to treatment, especially in Colombia, where regulation of over-the-counter antibiotics is weak [28].

On the other hand, no significant differences between colonization in hemodialysis patients and their household contacts were observed, unfortunately, the power of our analyzes was not sufficient due to the limitation of the number of patients. Nevertheless, this suggests that the colonization in this population is not only related to the hospital environment, similar to that reported by Bettin et al., [23]. In addition, it was found that factors such as hospitalization (PR 0.85 –CI 0.46–1.56 p = 0.600) and the consumption of beta-lactam antibiotics in the last year (PR 1.09—CI 0.59–2.01 p = 0.780) were unassociated with colonization by *S*. *aureus*, which coincides with what was observed by Yan et al. in a population of healthy adults in northern China, where there was no association between these factors and colonization [29].

About the factors associated with colonization, we observed that sharing a personal article, like towel, and a positive overcrowding index were associated with colonization, which have been described as risk factors because they allow closer and more frequent contact between people living at home [30]. On the other hand, home care service act as a protective factor, which is consistent with a study where only 2% of the patients attended with home care service had autochthonous colonization by *S*. *aureus* compared to 15.3% of the patients who were colonized upon admission to this service [31]. This may be due to the high consumption of antibiotics by patients and the proper application of personal protection measures by health workers [32].

Regarding molecular typing, a great diversity of *spa*-types was found, especially in MSSA, mainly in household contacts, but also in patients. Only two clonal complexes were observed in the MRSA isolates, of which 83.33% (n = 10) of the isolates belonged to CC8, which harbored IV SCC*mec* type. This has been described as a successful community clone that has caused high-frequency hospital infections [33]. Meanwhile, 11 different clonal complexes were observed in the MSSA isolates, where CC30 and CC45 stand out, for which a successful evolution to colonize humans has been suggested, and which also share a genetic relationship with successful MRSA CC30 clones [34]. Likewise, this agrees with that previously reported in the population: in MSSA, there is greater heterogeneity of clonal complexes [35]. These colonizing clones have been previously detected and reported colonizing children in Colombia [36] and are widely disseminated in Europe, South America, and the USA [35, 37].

Related to pets, colonization was found in 14% of the animals evaluated, most of them MRSA, similar to that reported in other studies [38]. Although no genetic relationship was found between isolates from pets and people, it is clear that dogs and cats can serve as reservoirs for this microorganism and be potential transmitters in the home due to the closeness and affection with which they are treated [38, 39]. This highlights the importance of taking them into account in the design and application of strategies to reduce colonization in homes.

As to the limitations in our study, colonization was evaluated in a single anatomical site in household contacts; however, the evaluated site is the main reservoir for this bacterium. Similarly, although colonization is an event that can change over time and requires several measurements, this is the first approach to colonization by *S*. *aureus* in the homes of hemodialysis patients and the community. As perspective for future studies, evaluating home surfaces and health workers of the renal unit could provide a better understanding of the transmission route of *S*. *aureus* among household members, also, the use of more complete molecular tools such as whole genome sequencing, could improve analyzes and establish a direction of transmission.

In conclusion, the frequency of colonization by *S*. *aureus* was high in both hemodialysis patients and their household contacts. The high genic relationship between isolates obtained demonstrates that this bacterium is being shared among household members and patients, increasing the risk of infections and showing the homes as an important source of colonization. On the other hand, the diversity observed shows that *S*. *aureus* acquisition can occur at the community level, starting from different sources not associated with the hospital. This study also demonstrates the importance of populations that circulate between the hospital and the community, who play an important role in the dynamics of the spread of this bacterium, and must be included in epidemiological education and control strategies.

## Supporting information

S1 Table Spa types in methicillin resistant and susceptible *S*. *aureus* isolates (MRSA-MSSA) from hemodialysis patients and their household contacts. (DOCX)

S1 Dataset Minimal dataset. (XLSX)
