# Supplementary material for: The remarkable genetic relationship between Staphylococcus aureus isolates from hemodialysis patients and their household contacts: Homes as an important source of colonization and dissemination
Source: PLoS One. 2022 Apr 19;17(4):e0267276. doi: 10.1371/journal.pone.0267276 (PMC9017883; doi:10.1371/journal.pone.0267276)
Supplement: S1 Table — (DOCX) [file pone.0267276.s001.docx]

**S1 Table:** ***Spa* types in methicillin resistant and susceptible *S. aureus* isolates (MRSA-MSSA) from hemodialysis patients and their household contacts.**

| *S. aureus* isolates | Participant | *spa-*type (Number of isolates) |
| --- | --- | --- |
| MRSA | Household contacts | t002 (1), t008 (1), t148 (2), t3092 (1), t4352 (1), t723 (1) |
|  | Patient | t002 (1), t008 (1), t1184 (1), t148 (1) |
| MSSA | Household contacts | t002 (1), t012 (1), t019 (1), t021 (2), t056 (2), t065 (2), t1077(1), t1156 (1), t1171(2), t1184 (1), t122 (1), t1236 (1), t1414 (1), t1451 (1), t201 (4), t209 (1), t214 (2), t2143 (1), t2155 (2), t2213 (1), t223 (2), t228 (2), t304 (1), t4315 (1), t4562 (1), t571 (2), t6463 (2), t831 (1), t922 (2) |
|  | Patient | t056 (1), t1077 (1), t1236 (3), t2155 (1), t228 (1), t2883 (2), t3249 (1), t330 (2), t3625 (2), t4315 (1), t6463 (1), t726 (1) |
